# Screening and expression validation of key proteins for secondary hair follicle growth in cashmere goats based on iTRAQ quantitative proteomics technology

**DOI:** 10.3389/fvets.2024.1441074

**Published:** 2024-10-15

**Authors:** Jiale Chang, Fanhua Meng, Ru Zhang, Juan Feng, Yujing Liu, Junjie Zhang, Zhaomin Liu, Jiayue Liang, Hongmei Xiao

**Affiliations:** ^1^College of Life Sciences, Inner Mongolia Agricultural University, Hohhot, China; ^2^Inner Mongolia Autonomous Region Key Laboratory of Biomanufacturing, Hohhot, China; ^3^Institute of Microbiology, Chinese Academy of Sciences, Beijing, China

**Keywords:** cashmere goat, secondary hair follicles, proteomics, western blot, immunohistochemistry, hair follicle cycle

## Abstract

**Background:**

The growth of secondary hair follicles (SHFs) in cashmere goats has periodic changes, including telogen, anagen, and catagen, during which proteins play important roles as the executor of life activities.

**Results:**

In this study, the skin tissues of cashmere goats at three different growth stages of SHFs were collected for proteome sequencing and validation experiments. Through protein differential expression analysis and time series analysis, FKBP prolyl isomerase 10 (FKBP10) and fibrillin 2 (FBN2) were screened as the key proteins for SHF cycle growth of cashmere goats, and albumin (ALB), collagen type I alpha 1 chain (COL1A1) and elastin (ELN) were predicted to be their interacting proteins. The results of quantitative real-time PCR (qRT-PCR), western blot, and immunohistochemistry experiments showed that the mRNA and protein expression levels of FKBP10, FBN2, COL1A1, ELN and ALB were higher in anagen and lower in telogen. They were all highly expressed in the outer root sheath of SHFs in anagen.

**Conclusion:**

FKBP10, FBN2, COL1A1, ELN, and ALB can promote the growth of SHFs in cashmere goats. This study lays the foundation for analyzing the growth cycle regulatory mechanism of SHFs in cashmere goats, and provides new ideas for further improving cashmere yield and quality.

## 1 Introduction

Cashmere goat (*Capra hircus*) is an important economic breed with the characteristics of drought resistance, strong disease resistance and tolerance to roughage (1, 2). Its cashmere is used as the preferred high-end textile raw material because of its soft texture, good thermal performance and air permeability, and enjoys the reputation of “soft gold” (3). In recent years, with the rapid development of the economy and the continuous reform and innovation of domestic cashmere products, it has become an inevitable trend to improve cashmere quality and yield (4). Cashmere goats have two different types of hair follicles. The primary hair follicles (PHFs) produce coarse hairs, which mainly provide mechanical protection for the animal bodies due to their thick and strong hair shaft. And SHFs produce cashmere, which have no medulla, are slender and soft and play a role in keeping animals warm (5, 6). SHF is a regenerative system with a complex developmental process, and its growth exhibits periodic changes with light intensity, which is divided into telogen, anagen, and catagen (7). Telogen is from February to March, anagen is from April to November, and catagen is from December to January of the following year (8). The structure of a hair follicle can be divided into five parts from bottom to top, including the hair bulb, keratin hyperplasia area, terminal sclerosis area, abscission area and funnel. The hair bulb surrounding the dermal papilla is the enlarged part of the end of the hair follicle (9). The hair papilla plays an important role in regulating the cyclical cycle of SHFs (10). In the early stage of hair follicle growth, dermal papilla cells proliferate and differentiate to form the inner root sheath and hair shaft. When hair follicles enter a vigorous growth period, their growth rate can reach the fastest. After anagen, all hair follicles enter catagen at similar times, and their activities gradually weaken over time (11). When hair follicles are ready to enter telogen from catagen, the inner root sheaths begin to undergo apoptosis. In telogen, the hair follicles are considered to be in a dormant state, and the hair shafts stop growing and begin to shed. Some studies have shown that telogen plays an important role in the hair follicle growth cycles, with its main function being to store energy, regulate and repair the skin microenvironment for hair follicle regeneration (12).

Proteome refers to the collection of all the corresponding proteins expressed in the genome, that is, the existence and activity of all proteins in cells, tissues, or organisms (13). Proteomics, which takes the proteome as the object, clarifies the expression and functional patterns of all proteins in organisms and has become a hot topic in life science in the post-genomic era. In order to explored the expression pattern of high mobility group nucleosome binding domain protein 1 (HMGN1) in hair follicles and whether the loss of HMGN1 can affect hair follicle development or cycle changes, Furusawa T. found that HMGN1 was expressed in the epidermal basal layer, outer root sheath and hair bulb in adult hair follicles, but not in the inner root sheath and hair shaft by proteomics technology. At the same time, he also found HMGN1 was expressed in some hair follicle stem cells (14). Yang et al. (15) screened for differentially expressed proteins in the skins of Yangtze River Delta white goats with type III and non type III wool, and identified four proteins. Among them, fibrinogen beta chain isoform 1 and ATP synthase beta subunit (ATB5B) were up-regulated in the skins with type III wool, while succinyl-CoA:3-ketoacid-coenzyme A transferase 1-mitochondrial-like and actin-cytoplasmic 1 (ACTB) were up-regulated in the skins with non type III wool, indicating that these four proteins play important roles in different aspects of hair follicle development. Through comprehensive analysis of cashmere goat skin transcriptome and proteome, Dai et al. (16) found that overexpression of thymosin beta 4 (Tβ4) in hair follicles could effectively increase cashmere yields. By the same method, Liu et al. (17) discovered that keratin (KRT) and collagen alpha families might play important roles in goat hair follicles and wool curvature development. Li et al. (18) confirmed that DSC2 gene is key to the woolly straight phenotype in goats through studying the differences of protein abundance in different fiber ranges from 18 sheep and goat wool samples. In the study on hair follicles in cashmere goats, Gao et al. (19) found that the expression levels of KRT1, KRT5, KRT25 and KRT71 were highest in anagen of hair follicles. In addition, studies have shown that *KRTAP28-1*, *KRTAP27-1*, *KRTAP24-1*, *KRTAP11-1*, *KRTAP15-1* and *KRTAP8-1* gene mutations are associated with cashmere fiber diameter (20–25), which has the value of molecular markers for improving cashmere diameter.

As a special biological resource, Albas white cashmere goats not only have delicious meat but are also recognized around the world for their unique cashmere. Many researchers try to understand the species at the molecular, cellular, and even individual levels, further improve its breeding, and expand the quantity and scale of breeding in order to improve cashmere yield and quality (26–29). Especially in recent years, many genes and proteins related to economic traits have been found through various omics analyses, which provide methods for the study on hair follicle growth and development in cashmere goats (30–33). However, research in the interactions between proteins regulating hair follicle growth and development, as well as the mechanisms related to hair follicle growth in cashmere goats, remains unresolved (34). In this study, skin samples of cashmere goats from three different periods were collected for proteome sequencing and validation experiments. Through sequencing data analysis, key proteins related to hair follicle growth of cashmere goats were screened, and their interacting proteins were predicted by bioinformatics methods. Meanwhile, the expression of key proteins and their interacting proteins was detected at the gene and protein levels by qRT-PCR, western blot, and immunohistochemistry technology. The purpose of this study was to lay the foundation for analyzing the regulation mechanism of SHF growth cycle of cashmere goats.

## 2 Methods

### 2.1 Animal and tissue collection

One centimeter square scapular skin samples of three 1.5-year-old Albas female cashmere goats with similar body weight and consistent feeding conditions according to standard cashmere goat husbandry practices were collected in anagen (September), catagen (December) and telogen (February) of SHFs. The skin samples from living body surface after hair removal and alcohol disinfection were collected by sterile surgical blades after the muscle tissue anesthetized with 1% pentobarbital sodium (Sinopharm Chemical Reagent Co., Ltd., China). Then Yunnan Baiyao powder (Yunnan Baiyao Group Co., Ltd., China) was applied to the wound sites to stop bleeding. The samples are divided into three parts, one part of them was placed in 4% paraformaldehyde (biosharp, Anhui, China) for paraffin section preparation, and the other two parts were placed in liquid nitrogen for sequencing and subsequent molecular experiments. All sample collections were conducted in accordance with the International Guiding Principles for Biomedical Research Involving Animals and with the approval of the Experimental Animal Welfare and Ethics Committee of Inner Mongolia Agricultural University.

### 2.2 Paraffin section embedding

The fixed skin tissues in paraformaldehyde were taken out, washed with PBS, then soaked in 50% alcohol for 30 min, 70% alcohol for 1 h, 80% alcohol for 1 h, and 95% alcohol for 1 h, and dehydrated with anhydrous ethanol I and II for 2 h, respectively. The samples were soaked in xylene and anhydrous ethanol (1:1) for 1 h and in xylene and paraffin (1:1) at 37°C overnight. After transparency with xylene, the samples soaked in soft wax and hard wax at 52°C and 56°C for 2 h, respectively, and embedded for tissue sections. One part was used for HE staining to observe the quality of sections, and the other part was used for immunohistochemical detection and microscopy photographing.

### 2.3 HE staining

Paraffin sections were deparaffinized in xylene for 10 min, twice. They were then hydrated through 95, 85, and 75% ethanol, followed by rinsing with deionized water. After stained with hematoxylin (LEAGENE, Anhui, China) for 10 min, the sections were rinsed with deionized water again, followed by differentiation in 1% acid alcohol (LEAGENE, Anhui, China), and another rinsed with deionized water. Eosin (LEAGENE, Anhui, China) staining was performed for 10 min. The sections were dehydrated through graded alcohols: 80, 85, 95%, and absolute ethanol, and in xylene twice for 10 min each time. Finally, neutral gum was applied to the sections, which were allowed to dry before being examined under a microscope (Eclipse Ti2-U, Nikon, Tokyo, Japan).

### 2.4 Protein extraction

Appropriate amount of frozen skin samples was taken and transferred into 1.5 mL centrifuge tubes. Samples were lysed by adding RIPA Lysis Buffer (Beyotime, Shanghai, China) and protease inhibitor PMSF (Beyotime, Shanghai, China) included to achieve a final concentration of 1 mM. Samples were subjected to ultrasonication on ice using a power setting of 80 W with cycles of 1 s on and off, for a total duration of 3 min. The lysates were centrifuged at room temperature at 12,000 × g for 10 min. The supernatant was carefully collected and subjected to a second centrifugation step under the same conditions. The protein concentration of supernatant was measured by BCA Protein Assay Kit (Solarbio, Beijing, China) and stored at −80°C after subpacking.

### 2.5 Proteome sequencing and data analysis

The different period total sample protein extracted by protein concentration and SDS-PAGE detection, were subjected to trypsin digestion and isobaric tag for relative absolute quantitation/tandem mass tags (iTRAQ/TMT) labeling. Then an equal amount of each labeled sample was mixed and separated by chromatography. Finally, the three group samples (anagen, catagen, and telogen) with 3 biological replicates in each group were analyzed by liquid chromatography-tandem mass spectrometry (LC-MS/MS). Chromatographic conditions: samples were uploaded at a flow rate of 400 nL/min to an analytical column Acclaim PepMap RSLC, 75 μm × 25 cm (RP-C18 ionopticks, Thermo Fisher) for separation. Mobile phase A phase: H_2_O-FA (99.9:0.1, v/v); mobile phase B phase: ACN-H_2_O-FA (80:19.9:0.1, v/v/v). Gradient elution conditions: 0–27 min, 2–21% B; 27–37 min, 21–42% B; 37–40 min, 42–95% B; 40–44 min, 95% B. Mass spectrometry conditions: capillary voltage of 1.5 kV, drying gas temperature of 180°C, flow rate of 3.0 L/min, primary MS scanning range of 100–1,700 m/z, ion mobility range of 0.6–1.6, collision energy range of 20–59 eV.

Based on the reliable proteins obtained by LC-MS/MS detection and database (Maxquant 1.6.17.0) search and compute, visualization of protein results after quality assessment and data normalization, the differential proteins were screened, and the screening conditions were set as Foldchange ≥2 (up-regulated protein expression was greater than or equal to 2 times, or down-regulated protein expression was less than or equal to 0.5 times) and *p* < 0.05. Correlation analysis, expression pattern clustering heat map, and Venn analysis were performed in R (Version 4.1.0). Various annotation information was extracted, and protein functions were mined from databases (UniProt, KEGG, GO, KOG/COG, etc.). At the same time, Gene Ontology (GO) analysis and Kyoto Encyclopedia of Genes and Genomes (KEGG) analysis^1^ of differential proteins obtained were performed. In the enrichment analysis, the Benjamini–Hochberg method was utilized for multiple test correction. The STRING (35)^2^ was used to upload the differentially expressed proteins in different periods to the String database for analysis. The visual protein–protein interaction (PPI) network was created by Cytoscape (Version 3.8.2), and the time series analysis of protein expression was performed by STEM (Version 1.3.13).

### 2.6 qRT-PCR validation

Total RNA was extracted from frozen cashmere goat skin samples using TRIzon Reagent (CWBIO, Jiangsu, China). The concentration and quality of the total RNA were assessed using NanoDrop 1000 (Thermo, Massachusetts, United States). The RNA extracted was reverse-transcribed into cDNA by the PrimeScript^™^ RT Master Mix kit (TAKARA, Dalian, China). The mRNA coding region sequences of the genes were retrieved from NCBI database,^3^ and the fluorescent quantitative specific primers of the genes were designed using Primer 5.0. The primer sequences are shown in Table 1. The relative expression levels of *ALB, COL1A1, FKBP10, ELN,* and *FBN2* genes were verified by qRT-PCR, and *GAPDH* (glyceraldehyde-3-phosphate dehydrogenase) was used as the reference gene. The reaction mix: TB Green Premix Ex Taq II (Tli RNaseH Plus) (2X) 10 μL, forward primer 0.8 μL, reverse primer 0.8 μL, cDNA 2 μL, and ddH_2_O 6.4 μL. Programs: initial denaturation (95°C 30 s), quantification (95°C 5 s, Tm 30 s) for a total of 45 cycles, melting (95°C 5 s, Tm 60 s, 95°C), and cooling (50°C 30 s).

**Table 1:** Primer sequences of candidate and reference genes.

Gene name	NCBI Reference Sequence	Primer sequences		Product length (bp)	TM (°C)
*COL1A1*	XM_018064893.1	F	GGTAGCCCTGGTGAAAATGGA	196	60
		R	CTTCACCCTTAGCACCCACA		
*ALB*	XM_005681744.2	F	GCGCTGATTTCCCTCTATTCA	1,910	60
		R	GCTGAGATGCTTGTGGTTGTG		
*FKBP10*	XM_005694317.3	F	ATGGAACAAGGAGGACACCG	1,133	60
		R	TGATGAAGGTGGAAAACTCCTC		
*FBN2*	XM_018050706.1	F	GCGTTACTGCACTGATGTTGA	885	60
		R	ATCCTGGCAAACCCTCTTGG		
*ELN*	XM_018040738.1	F	GGAAAGTGCCAGGAGTGGG	253	60
		R	TAAGGCAGTCCATAGCCACC		
*GAPDH*	XM_005680968.3	F	CTTCAACAGCGACACTCACTCT	122	60
		R	CCACCACCCTGTTGCTGTA		

### 2.7 Western blot

The target protein separated by SDS-PAGE electrophoresis was transferred to a polyvinylidene fluoride (PVDF) membrane soaked in methanol, and the blocking solution was added for shaking 1 h on a shaker. Then the PVDF membrane was washed with TBST 3 times. ALB, COL1A1, FKBP10, ELN, FBN2, and GAPDH were incubated overnight with primary antibodies (Bioss, Beijing, China). After PVDF membrane was washed with TBST 3 times again, the secondary antibody (GeneTex, United States) was added and incubated for 2 h. Followed by washing the PVDF membrane with TBST, and ECL luminescent solution (Servicebio, Wuhan, China) was applied to the membrane and imaged in an imaging device (ImageQuant LAS 500, GE Healthcare, Piscataway, New Jersey, United States).

### 2.8 Immunohistochemistry

Paraffin sections were baked at 65°C and then deparaffinized in xylene. They were subsequently hydrated through a graded series of ethanol: absolute ethanol, 95, 85, and 75% ethanol, followed by immersion in distilled water. The sections were then immersed in citrate buffer and microwaved for 8 min, followed by cooling the sections to room temperature. After cooling, they were washed with PBS, treated with 3% hydrogen peroxide for 10 min, and rinsed with PBS. The sections were incubated with 5% sheep serum at room temperature for 20 min. Primary antibody (Bioss, Beijing, China) was added, and the sections were incubated overnight at 4°C. After incubation, the sections were incubated with secondary antibody (GeneTex, United States) at 37°C in the dark for 30 min and washed with PBS. DAB working solution (Solarbio, Beijing, China) was added for color development. Counterstaining was performed with hematoxylin for 1–3 min, followed by washing with distilled water, differentiation with acid alcohol, and washing with PBS. They were then dehydrated through a graded series of ethanol: 70, 80, 90, 95%, and absolute ethanol. Sections are dipped twice in xylene. Neutral gum was applied to the sections, which were allowed to dry before examination under a microscope.

### 2.9 Statistical analysis

The relative expression levels of *ALB, COL1A1, FKBP10, ELN,* and *FBN2* genes were calculated using the 2^−∆∆Ct^ method. Data were analyzed using one-way ANOVA with SPSS (Version 25), followed by multiple comparisons using the Duncan method, and graphs were generated using GraphPad Prism (Version 9.5). *p* < 0.05 was considered statistically significant.

## 3 Results

### 3.1 Quality of tissue sections

**Figure 1:**
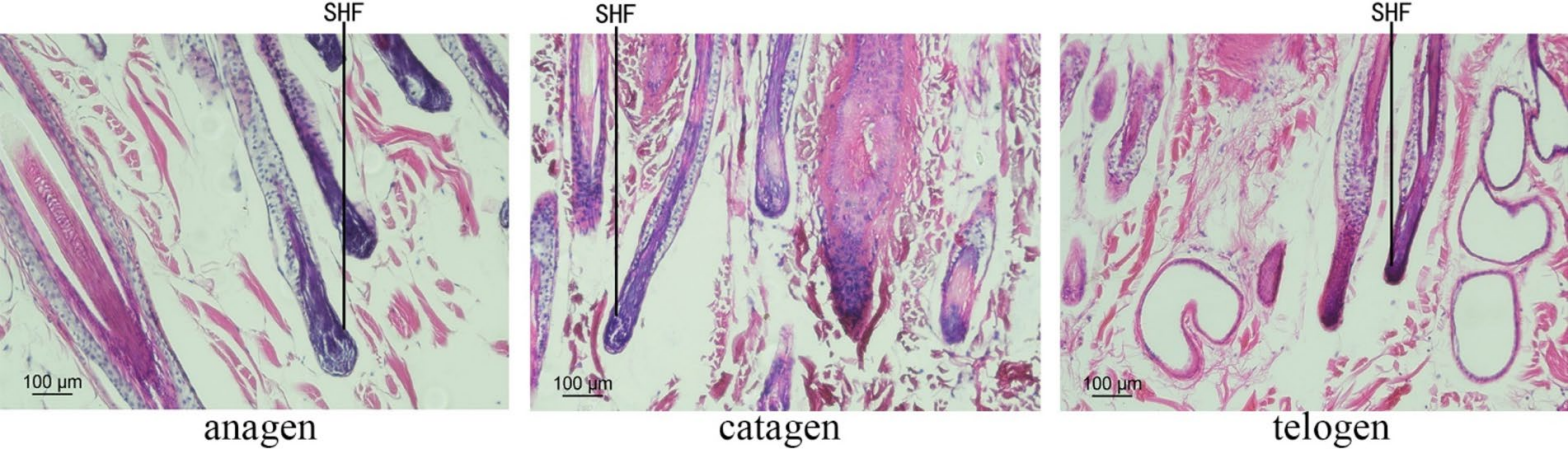
SHF morphological characteristics of cashmere goats.

The skin sections of Inner Mongolian Cashmere goats at different growth stages prepared were stained with hematoxylin and eosin (Figure 1). The growth cycles of PHFs in cashmere goats is different from that of the SHFs. In anagen, SHFs were round and full, indicating the active growth stage of the follicles. Dermal papillae were present in SHFs. In catagen, SHFs exhibited signs of atrophy. In telogen, further contraction of SHFs was observed, with reduced growth activity. The distribution of SHFs with clear outlines at different stages was suitable on the slices, showing that the section quality was better to can be used for subsequent experiments.

### 3.2 Principal component analysis

**Figure 2:**
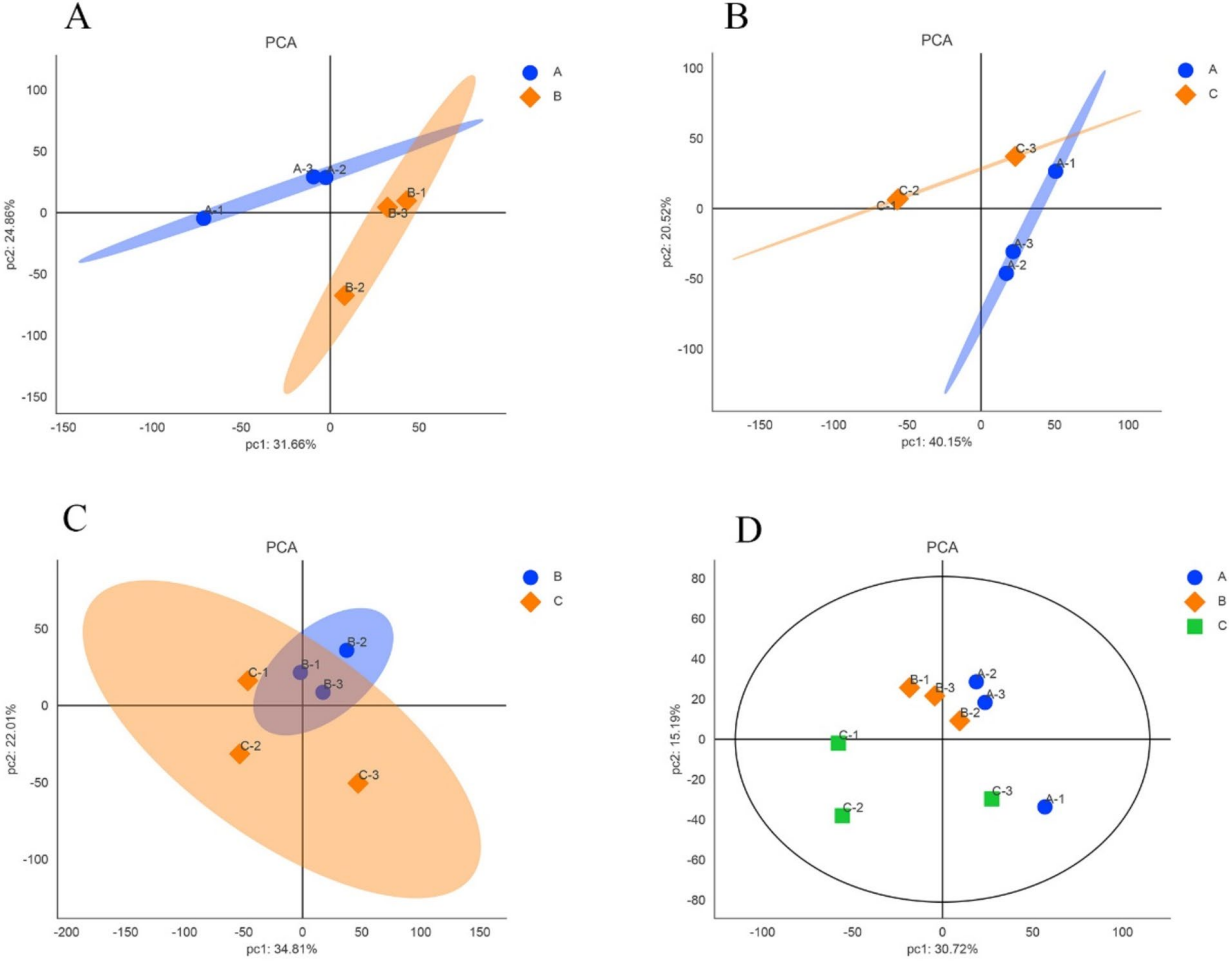
Principal component analysis of samples in different growth periods of SHFs in cashmere goats. **(A)** anagen and catagen **(B)** anagen and telogen **(C)** catagen and telogen **(D)** anagen, catagen and telogen. A, B, and C represent anagen, catagen, and telogen. In each figure, each point represents one replicate of an experimental group.

The expression levels of reliable proteins obtained from raw data through database retrieval and screening were subjected to principal component analysis (PCA). The PCA analysis results were shown in Figure 2. The more points clustered, the higher similarity between the samples, and the more points discrete, the lower similarity between the samples. As shown in Figures 2A,B, the confidence zones of the anagen samples and the other two period samples overlap less, and there are large differences between them. In Figure 2C, although there are differences between the catagen and the telogen samples, the reason for the larger overlap between them comparing with the telogen and the anagen samples may be due to the short time interval between catagen and telogen. Figure 2D showed that the three samples in anagen and catagen were more concentrated, indicating that there is little difference within the group, and the samples showed better correlation and similar of protein composition. The three samples in telogen are relatively dispersed, which may be due to individual differences in telogen.

### 3.3 Differential protein screening and specificity analysis of cashmere goat SHFs at different growth stages

**Figure 3:**
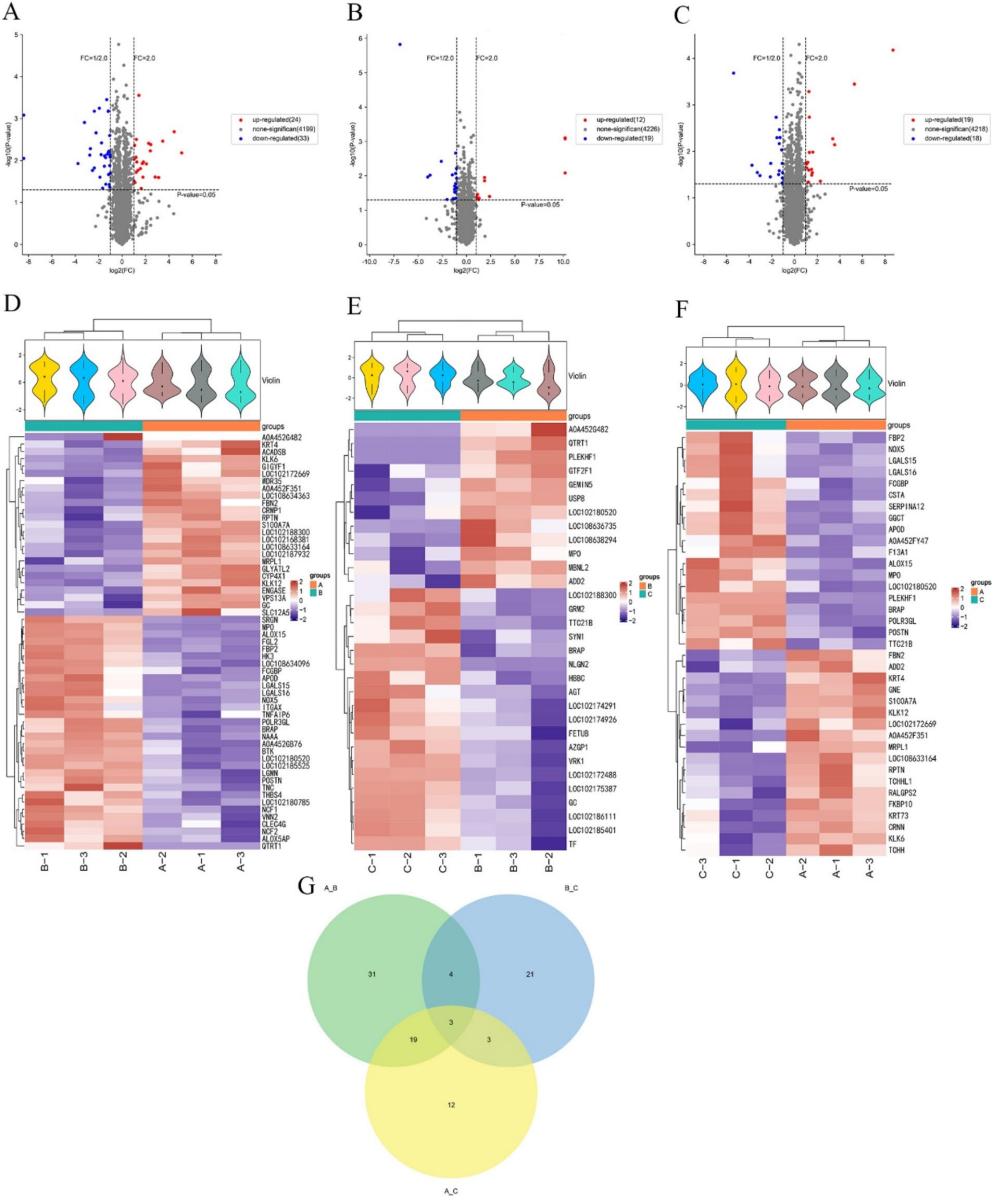
Screening of differential proteins in the skins of cashmere goats at different stages. **(A)** Volcano map of cashmere goat skins differential proteins in anagen and catagen. **(B)** Volcano map of cashmere goat skins differential proteins in catagen and telogen. **(C)** Volcano map of cashmere goat skins differential proteins in anagen and telogen. **(D)** Cashmere goat skins in anagen and catagen differential protein cluster analysis heat map. **(E)** Cashmere goat skins in catagen and telogen differential protein cluster analysis heat map. **(F)** Cashmere goat skins in anagen and telogen differential protein cluster analysis heat map. “+” represents the median. The vertical axis is the protein expression level, red indicates higher protein expression, and blue indicates lower protein expression. **(G)** Venn diagram of differential proteins. The numbers represent the number of differential proteins. A, B, and C represent anagen, catagen, and telogen.

The proteins expressed in three different periods were screened separately. In anagen and catagen, there were 57 differentially expressed proteins, of which 24 were up-regulated proteins and 33 were down-regulated proteins. The significantly up-regulated proteins were KLK6, FBN2, GLYATL2, LOC102188300, CYP4X1, LOC102172669, etc., and the significantly down-regulated proteins were NAAA, QTRT1, A0A452G482, LGALS15, LOC102180520, BRAP, etc. (Figures 3A,D). In catagen and telogen, there were 31 differential proteins, of which 12 were up-regulated proteins and 19 were down-regulated proteins. The significantly up-regulated proteins were MBNL2, PLEKHF1, QTRT1, USP8, GTF2F1, ADD2, etc., and the significantly down-regulated proteins were NLGN2, TTC21B, GRM2, BRAP, GC, SYN1, etc. (Figures 3B,E). In anagen and telogen, there were 37 differentially expressed proteins, of which 19 were up-regulated proteins and 18 were down-regulated proteins. The significantly up-regulated proteins were LOC102172669, LOC108633164, KRT4, S100A7A, FBN2, CRNN, etc., and the significantly down-regulated proteins were A0A452FY47, LGALS15, TTC21B, POLR3GL, LGALS16, LOC102180520, etc. (Figures 3C,F).

The screened differentially expressed proteins in different periods were made into a Venn diagram (Figure 3G). Figure 3G shows that a total of 93 differential proteins were enriched in three periods. The largest number of differential proteins, 57, were found in anagen and catagen. The least number of differential proteins was found in catagen and telogen, with only 31. There were 31 differential proteins specific to anagen and catagen, 21 differential proteins specific to catagen and telogen, and only 12 differential proteins specific to anagen and telogen. Three differential proteins among anagen, catagen and telogen were screened, namely BRAP, MPO and LOC102180520.

### 3.4 Differential protein Gene Ontology and Kyoto Encyclopedia of Genes and Genomes analysis

**Figure 4:**
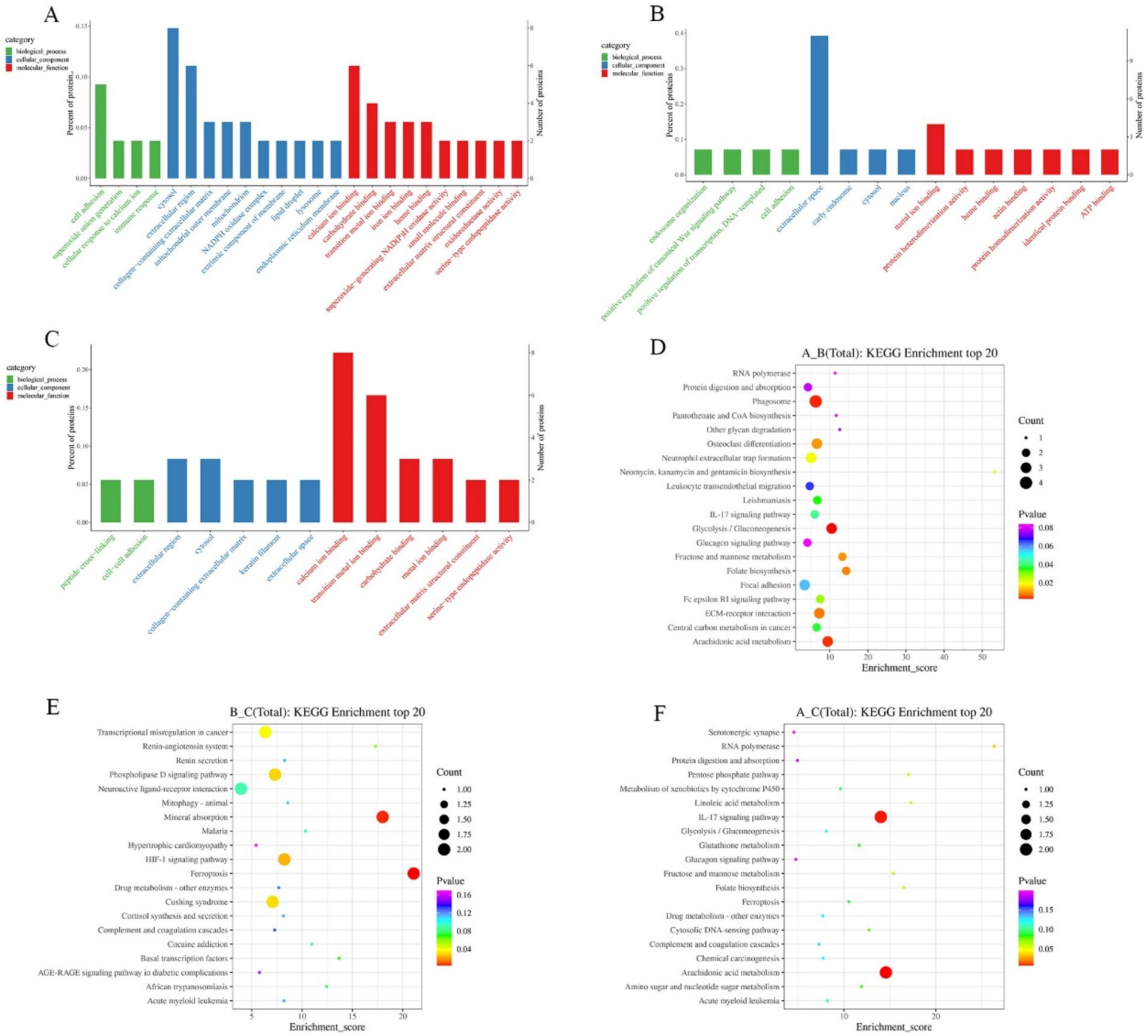
GO and KEGG enrichment analysis of differential proteins in different periods. **(A)** GO enrichment analysis in anagen and catagen. **(B)** GO enrichment analysis in catagen and telogen. **(C)** GO enrichment analysis in anagen and telogen. **(D)** KEGG enrichment analysis in anagen and catagen. **(E)** KEGG enrichment analysis in catagen and telogen. **(F)** KEGG enrichment analysis in anagen and telogen. The larger the bubble, the greater the number of differential proteins contained. The bubble color changes from red to green to blue-purple, and the smaller *p*-value, the greater the significance.

In order to comprehensively and systematically describe the functions of differential proteins in cells and organisms, the GO entries corresponding to the number of differential proteins greater than 1 were screened through GO annotation. Analysis was performed according to biological process (BP), molecular function (MF), and cellular component (CC). GO terms were ranked from the largest to the smallest based on their-log10(*p*-value), and the top 10 terms within each category (biological process, molecular function, and cellular component) were selected. The differential proteins in growth phase and degenerative phase mainly involved in the biological process of cell adhesion; in the cellular component, they were enriched in the cytosol, extracellular region, and other components; in terms of molecular function, they mainly involved in calcium ion binding, carbohydrate binding, and transition metal ion binding (Figure 4A). The differentially expressed proteins in catagen and telogen mainly involved in four biological processes: endosome organization, positive regulation of canonical Wnt signaling pathway, positive regulation of transcription, and cell adhesion. In the cellular component, they mainly were enriched in the extracellular space; in terms of molecular function, they involved in metal ion binding and other functions (Figure 4B). The differential proteins in the growth phase and the resting phase mainly involved in the biological processes of peptide cross-linking and cell adhesion. In the cellular component, they mainly were enriched in extracellular region, cytosol, and other components; in terms of molecular function, they mainly involved in calcium ion binding, transition metal ion binding, etc. (Figure 4C). Through the analysis of the GO categories of differential proteins across three periods, it was discovered that these proteins were predominantly enriched in the functions related to cell adhesion, cytosol, extracellular region, calciumion binding and carbohydrate binding. Differential protein enrichment on cell adhesion indicates the importance of cell–cell interactions in the hair follicle growth cycles. Differential protein enrichment on the cytosol and extracellular region indicates the cytosol and extracellular region as crucial sites for signal transduction and material exchange during the hair follicle cycle transition. Differential protein enrichment on calciumion binding may shed light on the pivotal role of calcium signaling in regulating the hair follicle growth cycles. Furthermore, differential protein enrichment on carbohydrate binding suggests that carbohydrate may play a role in structural support and signal transduction in regulating the hair follicle growth cycles. This not only underscores the central role of these biological processes and functions in regulating the hair follicle growth cycles, but also hints at underlying mechanisms that persist throughout the lifespan of the hair follicle.

Pathway analysis of differential proteins was performed using the KEGG database, and the significance of differential protein enrichment in each pathway entry was calculated by hypergeometric distribution test. Pathways were ranked from the largest to the smallest based on their-log10(*p*-value), and the top 20 pathways were selected for analysis. In anagen and catagen, MPO, NCF1, THBS4, and NCF2 were enriched in the phagosome pathway. Proteins enriched in glycolysis/gluconeogenesis pathway were A0A452DP35, FBP2, and HK3; proteins enriched in the arachidonic acid metabolism pathway were ALOX15, LOC102172669, and LOC102187932 (Figure 4D). LOC102172488 and TF were enriched in the mineral absorption pathway in catagen and telogen; lOC102172488 and TF were enriched in the ferroptosis pathway. AGT and GRM2 were enriched in the phospholipase D signaling pathway. LOC102172488 and TF were enriched in the HIF-1 signaling pathway (Figure 4E). At the growing and resting stages, S100A7A and LOC108633164 were enriched by the IL-17 signaling pathway, and ALOX15 and LOC102172669 were enriched in the arachidonic acid metabolism pathway (Figure 4F).

### 3.5 Differential protein PPI and time series analysis

**Figure 5:**
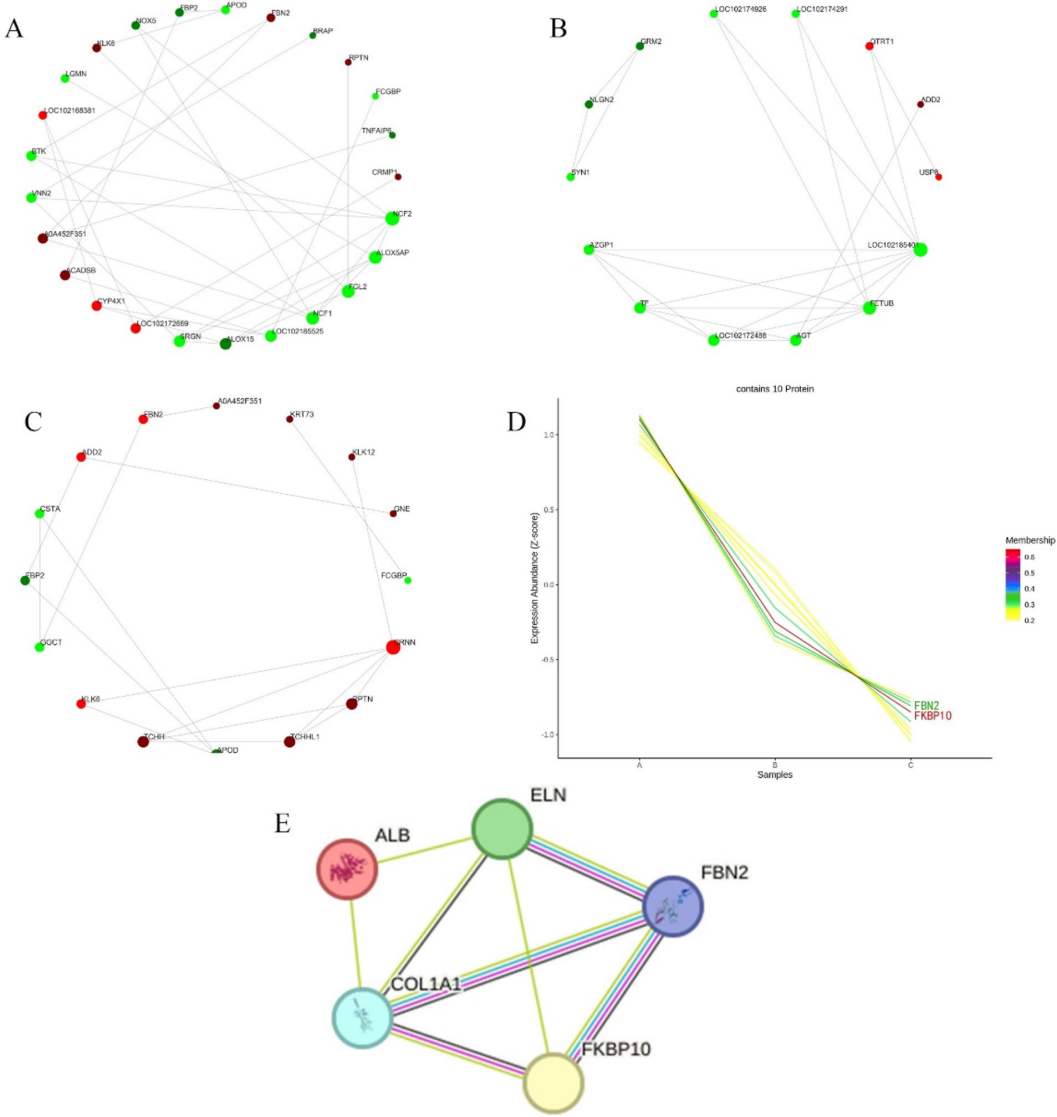
Differential protein interaction network and time sequence analysis. **(A)** Differential proteins interactions in anagen and catagen. **(B)** Differential protein interactions in catagen and telogen. **(C)** Differential protein interactions in anagen and telogen. In each figure, the red circle represents the up-regulated proteins; the green circle represents the down-regulated proteins; and the circle size represents the degree of connection. **(D)** Differential protein time series analysis. A, B, and C represent anagen, catagen, and telogen. Shades of color represent the degree of match. **(E)** Node proteins interaction network. Light blue and pink lines represent known interactions; blue comes from the selected database, and pink represents experimental determination; green, red, and dark blue lines represent predictive interactions; green represents gene neighborhoods; red represents gene fusion; and dark blue represents gene co-occurrence; the yellow line segment represents those mentioned in the literature; and the black line segment represents co-expression.

The differential proteins were analyzed in the STRING database to obtain the interaction relationship of the differential proteins. Then the interaction network visualization map of the top 25 node proteins with node connectivity was drawn by the Python software package “network.” There were 9 proteins up-regulated at the growth and regression stages, and they were LOC102172669, CYP4X1, ACADSB, A0A452F351, LOC102168381, KLK6, FBN2, RPTN, and CRMP1 according the interaction strength with other proteins. There were 16 down-regulated proteins, and the order of interaction with other proteins from strong to weak was NCF2, ALOX5AP, FGL2, NCF1, LOC102185525, ALOX15, SRGN, VNN2, BTK, LGMN, NOX5, FBP2, APOD, BRAP, FCGBP, and TNFAIP6 (Figure 5A). In telogen and catagen, 3 proteins up-regulated interacting with other proteins from strong to weak was QTRT1, ADD2, and USP8, and 11 proteins down-regulated interacting with other proteins from strong to weak were LOC102185401, FETUB, AGT, LOC102172488, TF, AZGP1, SYN1, NLGN2, GRM2, LOC102174926, and LOC102174291 (Figure 5B). At the growing and resting stages, 11 proteins up-regulated interacting with other proteins from strong to weak were CRNN, PPTN, TCHHL1, TCHH, KLK6, ADO2, FBN2, A0A452F351, KRT73, KLK12, and GNE. While 5 proteins were down-regulated, and the order of interaction with other proteins from strong to weak was APOD, GGCT, FBP2, CSTA, and FCG8P (Figure 5C).

Using Short Time-series Expression Miner (STEM) software, the time series analysis of differential protein expression in anagen, catagen and telogen was carried out, and then the corresponding change trend model was constructed. Cluster that matched the trend of highest expression during anagen, followed by the catagen, and lowest in telogen were selected, as shown in Figure 5D. The number of lines in Figure 5D represents the final number of proteins matched to this cluster. The membership value represents the degree of each protein fitting into this cluster. The larger membership values, the better fit. Finally, 10 proteins were screened, including FKBP10, FBN2, GNE, TCHH, KRT4, RALGPS2, TCHHL1, CRNN, KRT73 and GTF2F1.

### 3.6 Prediction of interaction proteins for key proteins

PPI analysis was performed on FKBP10 and FBN2 screened by the STRING online protein database. The number of protein network nodes was 5, and the average number of nodes was 3.2. The results showed that the interaction proteins of FKBP10 and FBN2 were COL1A1 and ELN. There is an interaction between FKBP10 and FBN2, and their homologues are co-expressed and interact with each other in other organisms. COL1A1 and FKBP10 are co-expressed in other organisms. COL1A1 interacts with both ELN and ALB (Figure 5E).

### 3.7 Expression of key proteins, interacting proteins, and their genes in SHF growth cycles

**Figure 6:**
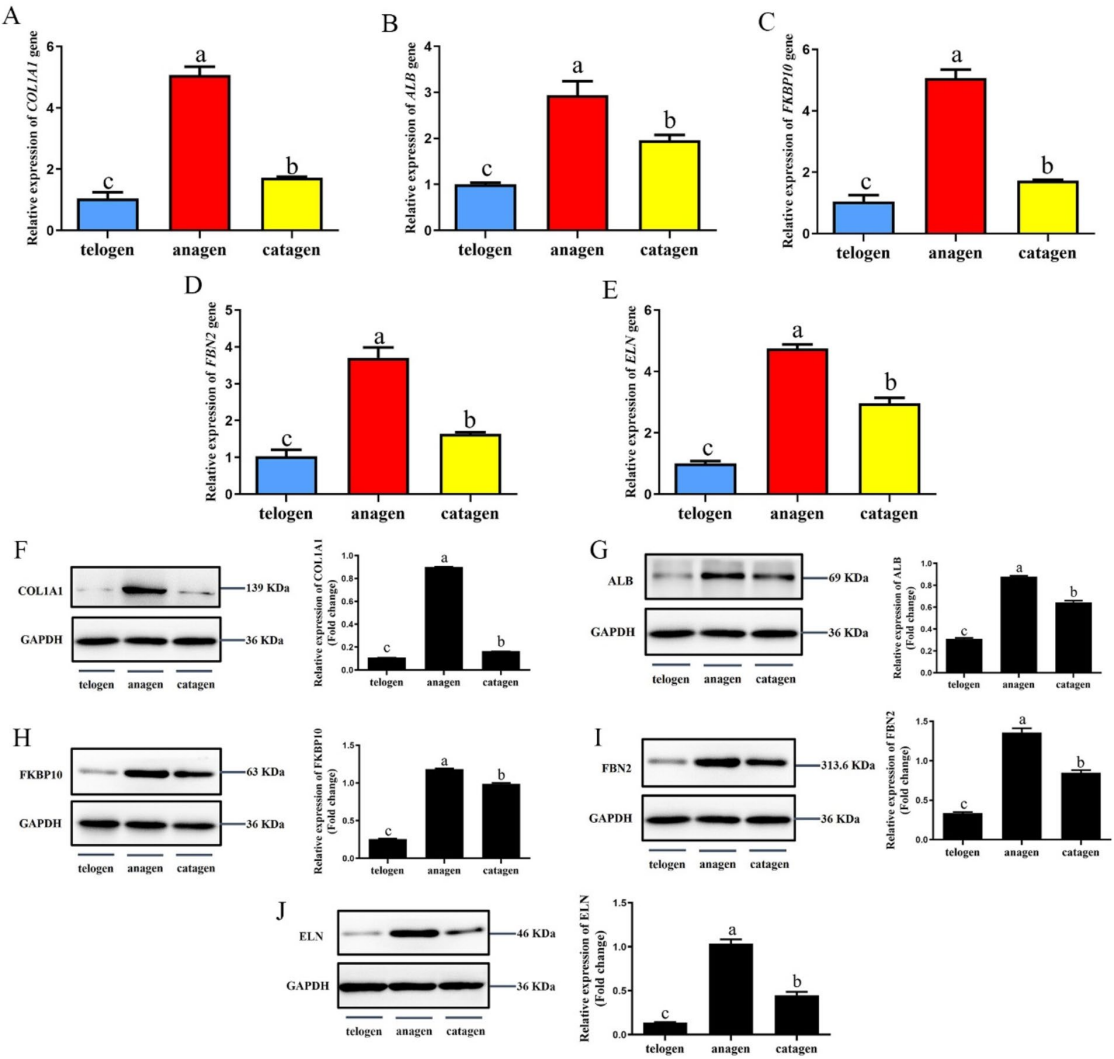
Expression of interaction proteins and their genes in the skins of SHFs in different growth periods. **(A)** The relative expression levels of *COL1A1* gene. **(B)** The relative expression levels of *ALB* gene. **(C)** The relative expression levels of *FKBP10* gene. **(D)** The relative expression levels of *FBN2* gene. **(E)** The relative expression levels of *ELN* gene. **(F)** COL1A1 expression and semi-quantitative analysis. **(G)** ALB expression and semi-quantitative analysis. **(H)** FKBP10 expression and semi-quantitative analysis. **(I)** FBN2 expression and semi-quantitative analysis. **(J)** ELN expression and semi-quantitative analysis.

The relative expression levels of *COL1A1*, *ALB*, *FKBP10*, *FBN2*, and *ELN* genes in different growth cycles of cashmere goat SHFs were significantly different (*p* < 0.05) (Figures 6A–E). They were expressed highest in the growing period, followed by the degenerative period, and lowest in telogen.

The protein expression levels of COL1A1, ALB, FKBP10, FBN2, and ELN in skins was detected by western blot (Figures 6F–J). The results showed that the expression trend of these proteins in the skins of different cashmere growth stages was consistent with the corresponding genes expression trend, and the protein expression level was the highest in the growth period, which was significantly higher than that in the regression period and telogen (*p* < 0.05).

### 3.8 The expression sites of key proteins and their interacting proteins in SHF growth cycles

The expression sites of FKBP10, FBN2, COL1A1, ELN, and ALB in SHFs was detected by immunohistochemistry. The signal intensity on the slices of different growth cycles was different.

**Figure 7:**
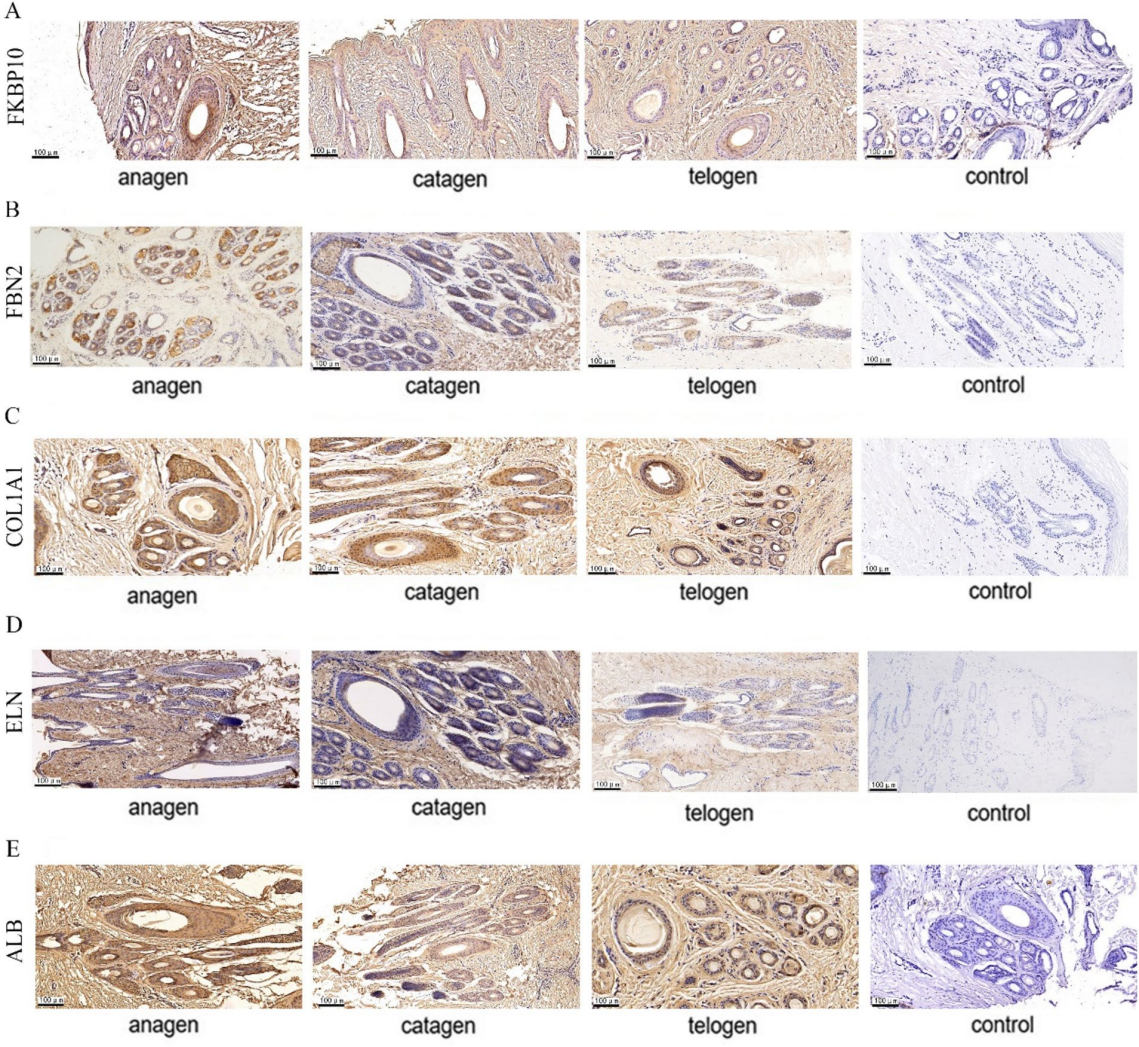
Five protein expression sites in SHFs at different growth stages. **(A)** FKBP10 immunohistochemistry **(B)** FBN2 immunohistochemistry **(C)** COL1A1 immunohistochemistry **(D)** ELN immunohistochemistry **(E)** ALB immunohistochemistry. Shades of brown indicate protein expression.

FKBP10 and FBN2 were significantly expressed in the outer and inner root sheaths of SHF growth period, and their expression levels in the inner root sheath were higher than that in the outer root sheath, while the expression level of two proteins in the inner root sheath and outer root sheath was significantly reduced in the degeneration period and significantly higher than that in telogen (Figures 7A,B). COL1A1 was significantly expressed in the outer root sheath and inner root sheath during SHF growth period. During the degeneration period, the protein was expressed more in the outer root sheath and less in the inner root sheath. The expression in the inner root sheath and outer root sheath of SHFs during the resting period was significantly reduced (Figure 7C). ELN is expressed in the outer root sheath of SHFs during the growth period, and it is only expressed in a small amount in the outer root sheath during the degeneration period, and no expression in the inner root sheath and the outer root sheath of SHFs in telogen (Figure 7D). The expression of ALB in the outer root sheath and inner root sheath was obvious in SHF growth period, and the expression in the inner root sheath was more than that in the outer root sheath. In catagen and telogen, ALB expression in the inner root sheath and outer root sheath was significantly reduced (Figure 7E).

In summary, these proteins are expressed in SHFs in different periods and highly expressed in the outer root sheath in anagen.

## 4 Discussion

The hair follicles of cashmere goats are divided into two types, namely PHFs and SHFs. SHFs produce unmyelinated cashmere, while PHFs produce medullated coarse hairs (36). Under the influence of *in vivo* and *in vitro* environments, as well as the skin microenvironment, hair follicles show strong self-regeneration ability and undergo periodic changes from anagen of rapid growth and development to catagen when hair follicle cells begin to apoptosis and then to telogen of a relatively static state (37). In anagen, SHFs are in an active stage of growth and development, and the length of fleece depends on the duration of this stage. The longer growth period, the longer the wool fiber produced by cashmere goats. In recent years, many researchers have utilized transcriptomics, genomics, and other omics approaches to investigate cashmere goat hair follicles. With the development of modern sequencing technologies, whole genome sequencing is able to examine sequence differences between populations, morphologies, and varieties of a large number of genes and gene families simultaneously (38). Li et al. (39) identified 135 genomic regions associated with cashmere fiber traits in cashmere goat populations through whole genome sequencing of 70 cashmere goats, including genes that may be involved in cashmere fiber production, such as *FGF5*, *SGK3*, *IGFBP7*, etc. Whole transcriptomics is becoming increasingly important due to the increasing research on non-coding RNAs and the boom in high-throughput sequencing. Liu et al. (40) used transcriptomic sequencing to study the mRNA-microRNA regulatory mechanisms of cashmere growth in cashmere goats under different photoperiods, revealing and validating the target relationships of differentially expressed genes and miRNAs. *BSDC1* and *RHBDF2* were targeted to miRNA-107-3p, and *ARSA* was targeted to miRNA-107-3p. *ALDH3A2* was targeted to miRNA-30b-3p. Xu et al. (41) adopted an integrated approach using transcriptomics, translatomics, proteomics, and metabolomics to identify substances associated with cashmere fineness, determining key regulators of cashmere fineness, including *PLA2G12A*, *KRT79*, and *prostaglandin B2*. During the periodic changes in hair follicle growth, many proteins involve in biological signal transduction, gene expression regulation, material and energy metabolism, cell cycle regulation, and other life activities through interactions with each other (16, 42–44). These proteins are trichohyalin (TCHH), KRT26, repetin (RPTN), cornulin (CRNN), tenascin-C (TNC), arachidonate-15-lipoxygenase (Alox15), SRY-box 18 (SOX18), matrix metalloproteinase-9 (MMP9), COL1A1, S100A7A etc. It is reported that TCHH was expressed in epithelial cells characterized by high mechanical strength, such as the inner root sheath cells of hair follicles. Its function was as an interfilament matrix protein by cross-linking with the head and tail ends of keratin intermediate filaments within the inner root sheath (45), and as a cross-linking enhancement protein, TCHH interacting with proteins such as RPTN, is crucial for the mechanical protection of the hair follicles. Through studying the expression patterns of specific genes of the hair follicles at different developmental stages in the inner root sheath of the embryonic of Tan sheep, Shi et al. (46) found that the expression level of *TCHH* gene was consistent with the pattern of wool crimping, and by affecting the morphology of the inner root sheath, it thereby affected the crimping of the wool of the beach sheep. Xu et al. (47) found that *KRT26* and *TCHH* genes were associated with cashmere fineness through studying the relationship between *KRT26* and *TCHH* gene polymorphisms and the production performance of Liaoning cashmere goat, which provided more theoretical basis for further research on cashmere fineness. *RPTN* involved in interactions with *TCHH* (48). *RPTN* was a member of the fusion protein family and is located on the epidermal differentiation complex on chromosome 1q21. The fusion protein family was associated with keratin intermediate filaments and is partially cross-linked to the cell envelope (CE) (49). In addition, CRNN of the fusion protein family is predominantly found in the upper spiny and granular layers of the epidermis and may be involved in the formation of the CE (50). Kim et al. (51) studied the anti-inflammatory role of *Alox15* in skin homeostasis. The authors knocked out *Alox15* in mice resulting in loss of hair follicle stem cells and an abnormal transition of dermal adipocytes to fibroblasts. Although the skin of *Alox15* knockout mice appeared to develop normally after birth, impaired skin barrier as well as hair loss was observed in adult mice. *TNC* was a marker for touch dome keratin-forming cells, which induced new hair follicles upon stimulation (52), and it was thought to be a regulator of WNT signaling in hairs and is required for Wnt/β-catenin signaling (53). SOX2 and SOX18 of SOX family proteins played important roles in determining hair follicle types, and Villani et al. (54) demonstrated that Sox18 regulates the normal differentiation of dermal papillae in all hair types. WNT5A and TNC are potential downstream effectors of SOX18 and were important for epidermal WNT signaling, and the expression of *WNT5A*, *MMP9* and *TNC* was significantly reduced in the skin of Sox18+/OP mice. Through RNA-seq of skin samples from E60 and E120 cashmere goat embryos as well as newly born cashmere goats, Gao et al. (55) showed that *TNC* was up-regulated as a key gene and expressed in HF initiation from E60 to E120. To explore the molecular mechanism of hair bending with growth, Liu et al. (17) identified *CRNN* and *TNC* as candidate genes for wool bending in Zhongwei goats. Tian et al. (56) showed hair growth is regulated by the *KRT4* gene interaction network in a study of vellus hair follicles in different seasons in Min pigs. Based on comprehensive analysis of the transcriptome and proteome of cashmere goat skins, Dai’s et al. (16) showed that KRT4 and tβ 4 may interact to mediate extracellular signal regulated protein kinase (ERK) signaling pathway, thereby affecting hair growth, promoting SHF development, and improving cashmere production. *KRTs* family showed strong correlation throughout the hair follicle development cycle in yak, where *KRT4* was associated with cell cycle regulation, hair follicle development, and proliferation of keratin-forming cells (57). Wu et al. (7) examined mRNA, lncRNA and their candidate genes in SHF development and cycling. It was determined that *S100A7A* is a key gene with importance in hair follicle development and cycling in the Jiangnan cashmere goat.

In this study, not only TCHH, KRT4, RALGPS2, CRNN, ALOX15, TNC, MMP9 and other proteins were screened, but also FKBP10, FBN2, GNE, TCHH1, KRT73 and GTF2F1, etc. were screened by analyzing the skin proteomics data of cashmere goats at different hair follicle growth stages. Therefore, we mainly verified the expression levels of FKBP10 and FBN2, and predicted their interaction proteins, ALB, COL1A1, and ELN. The experimental results were consistent with the sequencing results by qRT-PCR, western blot, and immunohistochemical verification at the gene and protein levels. They were highly expressed in the growth period of hair follicles, lowly expressed in telogen, and mainly expressed in the outer root sheath of SHFs. It shows that these five proteins promote hair follicle growth through different interactions. However, the specific way they interact in SHFs and their effects on hair follicle cells are still unclear. It is only found in the researches of others in other fields. FKBP10 is a member of the FK506-binding proteins (FKBPs). FKBPs are a class of highly homologous proteins widely expressed in eukaryotic cells that can assist transcription factors to regulate gene expression, participate in protein folding, modification, and transport, and have important functions. For example, studies on the relationship between *FKBP10* and *COL1A1* have shown that *FKBP10* silencing can significantly reduce the mRNA level of *COL1A1*. It is speculated that *FKBP10* silencing will change the quantity or cross-linking of extracellular matrix (58). Both of them can regulate Wnt/β-catenin and PI3K/AKT pathways related to cell proliferation and abnormal cell migration (59), promote the formation of cancer cells or participate in the occurrence of tumors through the interaction of cell adhesion molecules and receptors (60, 61), and regulate the fracture repair of osteoporosis (62). In the study of a mouse lung cancer model, it was found that the low expression of the FKBP10 protein could inhibit the occurrence of lung cancer. When endoplasmic reticulum stress occurred, FKBP10 protein was significantly down-regulated, and the expression of CyclinD1 protein decreased, which blocked the cell cycle and inhibited cell proliferation (63). ELN is an elastin, which is a common extracellular matrix component in connective tissue and plays an important role in maintaining tissue structure and function. The elastic fibers of skin tissue are loose network structures. The main components of elastic fibers are elastin and microfibers, and the formation of elastic fibers requires a variety of extracellular matrix proteins to be synthesized in a specific temporal and space order, while microfibers are composed of fibrillar protein (FBN), of which FBN2 is the main component (64, 65). In the early stage of tissue development, FBN1 and FBN2 synthesize microfiber bundles with a diameter of about 10 nm in the extracellular matrix in the form of N-terminal and C-terminal binding (66, 67), which is the basis for the final formation of the fiber bundle morphology of elastic fibers, binding to fibronectin (FN) and being fixed around the cell (68, 69). Mutations in the *FBN2* gene are closely related to hereditary connective tissue diseases, such as congenital contractural arachnodactyly (CCA), macular degeneration (MD), and myopathy, etc. (70). Studies have found that in the early stage of tissue development, the content of microfibers is high, and after tissue development, the content of ELN is also high, indicating that ELN can bind to FBN2 and participate in the assembly of microfibrils and the regulation of growth factors (71, 72). Due to it can participate in many physiological processes such as promoting extracellular matrix synthesis, cell proliferation, and migration (73–76), in recent years, through gene co-expression analysis and proteome sequencing of cashmere goats, *COL1A1* has been found to be associated with SHF growth (77, 78). It is believed that it can enhance the aggregation of dermal papilla cells and promote the growth of SHFs by affecting cell adhesion and movement (42). Cashmere fineness is a critical factor in determining cashmere quality. Wang et al. (28) used single-cell RNA sequencing to identify 13 skin cell types in Liaoning cashmere goats. Through cell trajectory analysis, the molecular changes during development were analyzed. SHF dermal papilla cells play a significant role in cashmere growth. It was found that COL1A1 is highly expressed in SHF dermal papilla cells. Zhao et al. (79) studied the molecular mechanism of hair follicle development through methylation and the whole transcriptome, proved the interaction between *E65*, *E85*, *E105*, and *E135* and DNA methylation, and studied the development stages and genes related to wool growth, laying a foundation for improving wool trait breeding.

In order to further understand the interaction between the five proteins and reveal their roles in the growth of cashmere goat hair follicles, the following experiments will be carried out through yeast two-hybrid and co-immunoprecipitation to explore the signaling pathways and interaction modes regulated by them. The function of the above proteins in secondary dermal papilla cells will be verified by overexpression and interference techniques.

## 5 Conclusion

Based on iTRAQ quantitative proteomics technology, we identified and screened differential proteins in skins of cashmere goat in anagen, catagen and telogen. There were 57 differential proteins screened in anagen and catagen, of which 24 were up-regulated and 33 were down-regulated in anagen. There were 31 differential proteins screened in catagen and telogen, of which 12 were up-regulated and 19 were down-regulated in catagen. There were 37 differential proteins screened in anagen and telogen, of which 19 were up-regulated and 18 were down-regulated in anagen. Through analysis, two key proteins and three interacting proteins of hair follicle growth in cashmere goats were identified, namely FKBP10, FBN2, COL1A1, ELN, and ALB. The expression levels of these five proteins in skins of cashmere goat were highest in anagen, followed by catagen, and lowest in telogen. They are highly expressed in the outer root sheath of SHFs during the growing period, which can promote the growth of SHFs.

## Data Availability

Data during the current study are available from the corresponding author on reasonable request. Requests to access these datasets should be directed to Hongmei Xiao: lhtdyx@126.com.

## Footnotes

^1^ https://cloud.oebiotech.com/#/bio/detail?number=7e90ec6d-9ef9-422a-8301-fc75b49970f3

^2^ https://string-db.org/

^3^ https://www.ncbi.nlm.nih.gov/
